# Is the implementation of a microbiological surveillance screening beneficial in a neonatal intensive care unit?

**DOI:** 10.1186/2194-7791-2-S1-A17

**Published:** 2015-07-01

**Authors:** I Schmeh, A Welk, T Schwanz, A Diefenbach, B Jansen, E Mildenberger

**Affiliations:** Department of Neonatology, University Medical Center of the Johannes Gutenberg University Mainz, Germany; Department of Medical Microbiology and Hygiene, University Medical Center of the Johannes Gutenberg University Mainz, Germany

## Background and aims

Bacteria that cause nosocomial infections have often been found to colonize the patient's skin, respiratory tract or gastrointestinal tract previously. In 2012 and 2013, the German Commission for Hospital Hygiene and Infectious Disease Prevention recommended a microbiological screening of infants on neonatal intensive care units. Multidrug-resistant bacteria (MDR) that should be considered in empiric antibiotic therapy, bacteria that cause invasive infections and bacteria that may elicit epidemic infections were regarded as target bacteria. The aim of this study was to evaluate the benefit of this kind of screening in clinical practice. We investigated the epidemiology and dynamic of colonization, determined the number of nosocomial infections caused by pathogens identified by the screening and analyzed the cases in which the preemptive adaptation of the empiric antibiotic therapy due to the detection of MDR was of proven benefit.

## Methods

This prospective study was conducted over a 22-month period from July 2012 to April 2014. We analyzed pharyngeal and rectal swabs once a week. Patient's characteristics and occurring nosocomial infections were recorded.

## Results

35% of the included 205 patients were colonized with target bacteria. 10% were colonized with MDR. *Enterobacter* spp. and *Klebsiella pneumoniae* were detected most frequently. 3/41 recorded nosocomial infections could be attributed to bacteria that were previously identified by the screening. 9 patients were treated with third line antibiotics as consequence of colonization with MDR. In only one infection MDR was identified as the causative pathogen.

## Conclusions

A benefit of the screening in terms of relevant preemptive adaptation of the empiric antibiotic therapy was proven only for 1/205 patients. In 3/41 infections the pathogens were previously identified by the screening. Negative implications may be an elevated use of third line antibiotics.

